# Supplementary material for: Expression of miRNAs Targeting ATP Binding Cassette Transporter 1 (ABCA1) among Patients with Significant Carotid Artery Stenosis
Source: Biomedicines. 2021 Jul 30;9(8):920. doi: 10.3390/biomedicines9080920 (PMC8406092; doi:10.3390/biomedicines9080920)
Supplement: Supplementary file 1 [file biomedicines-09-00920-s001.zip › biomedicines-1273825 supplementary.pdf]

Supplementary Material

The surgical procedure of carotid endarterectomy (CEA)

The CEA procedure used has been previously detailed [1-3]. The surgical procedures were carried out under general anesthesia with endotracheal intubation. Systemic heparinization was given to all patients (5,000 IU, intravenously) before carotid clamping. In this study, an outlying internal shunt was inserted during CEA in all patients. Endarterectomy with patch angioplasty was performed in the standard fashion with optical 3.5 x power magnification and Prolene 5/0 and 6/0 continuous sutures. Tacking sutures to secure the distal intima was performed if needed.

References

1. Kim, J. H., Cho, Y. P., Kwon, T. W., Kim, H., and Kim, G. E. “Ten-year comparative analysis of bovine pericardium and autogenous vein for patch angioplasty in patients undergoing carotid endarterectomy.” *Ann Vasc Surg* 26, no. 3 (2012): 353-8.

2. Kim, A., Kwon, T. W., Han, Y., Kwon, S. U., Kwon, H., Noh, M., and Cho, Y. P. “Clinical outcomes of staged bilateral carotid endarterectomy for bilateral carotid artery stenosis.” *Ann Surg Treat Res* 89, no. 5 (2015): 261-7.

3. Jeong, M. J., Kwon, H., Jung, C. H., Kwon, S. U., Kim, M. J., Han, Y., Kwon, T. W., and Cho, Y. P. “Comparison of outcomes after carotid endarterectomy between type 2 diabetic and non-diabetic patients with significant carotid stenosis.” *Cardiovasc Diabetol* 18, no. 1 (2019): 41.

Supplementary Figure

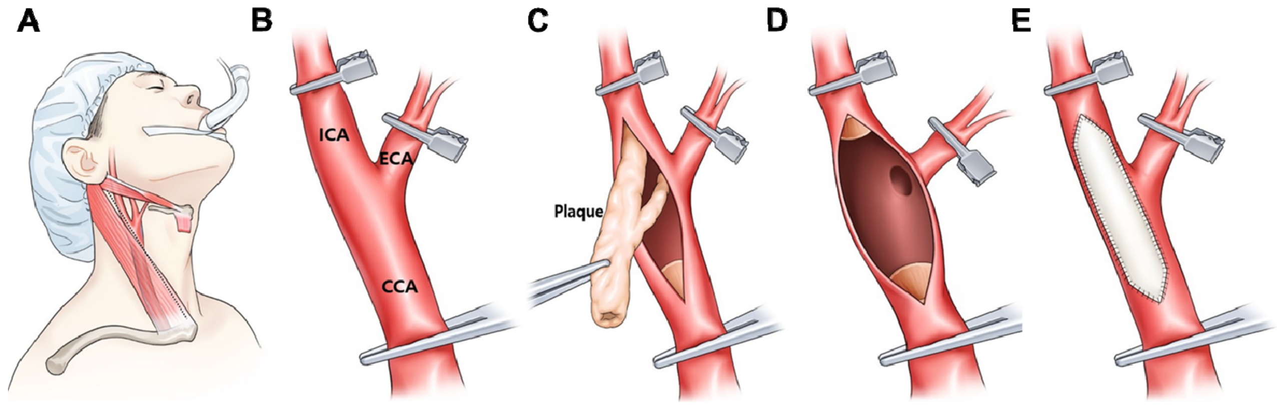

Supplementary Figure S1. Schematic representative figures of the surgical procedure of carotid endarterectomy. (A) The patient's neck is hyperextended with the chin turned away from the operative side. The incision runs parallel to the anterior border of the sternocleidomastoid muscle. (B) The common (CCA), internal (ICA), and external (ECA) carotid arteries are exposed. (C) After vertical arteriotomy, endarterectomy procedure is begun, and carotid plaque is removed. (D) The endarterectomy is ended at the appropriate points on the CCA and ICA. (E) The arteriotomy is closed with a patch.

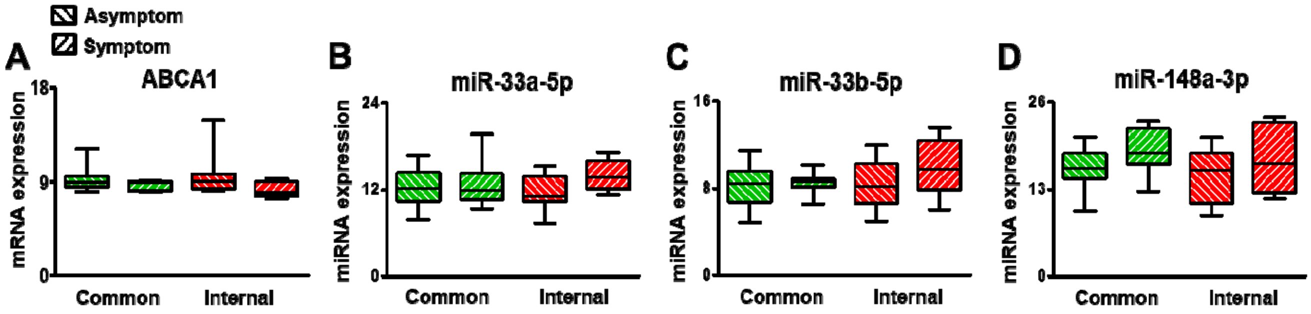

Supplementary Figure S2. Expression of ABCA1 and its target miRNAs in carotid artery stenosis with asymptomatic and symptomatic patients. (A) mRNA expression of ABCA1 by qRT-PCR. Expression of miR-33a-5p (B), miR-33b-5p (C), and miR-148a-3p (D) by qRT-PCR. Mean ± SD. Common, common carotid artery; Internal, internal carotid artery.
